# Long term survival results for gastric GIST: is laparoscopic surgery for large gastric GIST feasible?

**DOI:** 10.1186/1477-7819-10-230

**Published:** 2012-10-31

**Authors:** Ki-Han Kim, Min-Chan Kim, Ghap-Joong Jung, Su-Jin Kim, Jin-Seok Jang, Hyuk-Chan Kwon

**Affiliations:** 1 Departments of Surgery, Dong-A University College of Medicine, 3-1 Dongdaeshin-Dong, Seo-Gu, Busan, 602-715, Korea; 2 Departments of Pathology, Dong-A University College of Medicine, Busan, Korea; 3 Departments of Internal medicine, Dong-A University College of Medicine, Busan, Korea

**Keywords:** Stomach, GIST, Laparoscopy, Survival

## Abstract

**Background:**

Recently, laparoscopic resection for relatively small sized gastric gastrointestinal stromal tumors (GISTs) has been widely accepted as minimally invasive surgery. However, no report on the long-term safety and efficacy of this surgery for large sized gastric GISTs has been published to date.

**Methods:**

Between July 1998 and January 2011, 104 consecutive patients who underwent resection for gastric GISTs were enrolled in this retrospective study. We assessed the clinicopathological characteristics, postoperative outcomes, patient survival, and tumor recurrence.

**Results:**

Of the 104 patients with gastric GISTs who were included in the study, there were 47 males and 57 females whose mean age was 59.8 years. Sixty-four patients (61.5%) had symptoms associated with tumor. *Ten patients included in the group 1, 49 in the group 2, 15 in the group 3a, 9 in the group 5, 14 in the group 6a, and 7 in the group 6b.* There was one minor complication and no mortalities. Recurrence was noted in 5 patients, with a median follow-up period of 49.3 months (range, 8.4 to 164.4). The 5-year overall and disease free survival rates of 104 patients were 98.6% and 94.8%, respectively. When comparing large tumor (5–10 cm) between laparoscopic and open surgery, there were statistically differences in age, tumor size, tumor location, and length of hospitalization. There were no statistical differences in the 5-year survival rate between laparoscopic and open surgery for large tumor (5-10cm).

**Conclusion:**

Laparoscopic surgery is feasible and effective as an oncologic treatment of gastric GISTs. Moreover, laparoscopic surgery can be an acceptable alternative to open methods for gastric GISTs of size bigger than 5 cm.

## Background

Gastrointestinal stromal tumors (GISTs) represent a rare but distinct histopathological group of intestinal neoplasms of mesenchymal origin. Their incidence is only 0.2% of all gastrointestinal malignancies [1]. Despite the development of a new chemotherapeutic agent, imatinib mesylate, surgery remains the only curative treatment for non-metastatic gastric GIST [2]. Resection with a negative margin should be performed. Lymphadenectomy is not necessary, because gastric GISTs rarely metastasize to the lymph node [3]. Wedge resection has been practiced in open and laparoscopic procedures, but recent National Comprehensive Cancer Network (NCCN) guidelines have not yet described definite indications for these options [4]. Currently, laparoscopic wedge resection is a good surgical option for gastric GIST and is an alternative to conventional open surgery. However, there has been controversy regarding tumor size in laparoscopic surgery for gastric GISTs [5]. Recent reports show that laparoscopic or laparoscopic-assisted resection may be used for small gastric GISTs [6]. However, no report on the long-term safety and efficacy of this surgery for large-sized gastric GISTs has been published to date, even though some publications showed its short-term feasibility for large gastric GISTs.

In this report, we present a retrospective review evaluating the clinicopathological characteristics, postoperative outcomes, patient survival, and tumor recurrence of gastric GISTs after surgical treatment. Moreover, we tried to confirm the safety and efficacy of laparoscopic surgery for gastric GISTs larger than 5 cm in size.

## Methods

### Patients’ evaluation and work up

We reviewed the medical records of 104 patients with gastric GISTs who underwent curative resection in Dong-A University College of Medicine between July 1998 and January 2011. Patients with unresectable metastasis or concurrent cancer other than gastric GIST were excluded. We made the diagnosis of gastric GIST by final pathology among the patients with gastric submucosal tumors which were found preoperatively by esophagogastroduodenoscopy (EGDS), abdominal computed tomography (CT), and endoscopic ultrasonography (EUS).

According to risk classification suggested by Miettinen *et al*., the gastric GISTs were divided into eight groups based on maximum tumor diameter and mitotic activity per 50 high-power fields (HPFs), as previously detailed (Table 1) [7]*.* Patient characteristics, measured perioperative parameters that included operation method and type of resection, operative times, length of hospitalization, complication, tumor recurrence, median follow-up periods, and patient survival were evaluated. Also, we compared the clinicopathologic characteristics and postoperative outcomes of patients with large tumors (5 to 10 cm) between laparoscopic and open surgery.

**Table 1:** Suggested guidelines for assessing the malignant potential of gastric GISTs of different sizes and mitotic activity

Benign	
	Group 1 (no larger than 2 cm, no more than 5 mitoses/50 HPF)
Probably benign (very low malignant potential)	
	Group 2 (> 2 cm and ≤ 5 cm, no more than 5 mitoses/50 HPF)
	Group 3a (> 5cm and ≤ 10 cm, no more than 5 mitoses/50 HPF)
Uncertain or low malignant potential	
	Group 4 (no larger than 2 cm, > 5 mitoses/50 HPF)
Low to moderate malignant potential	
	Group 3b (> 10 cm, no more than 5 mitoses/50 HPF)
	Group 5 (> 2 cm and ≤ 5 cm, > 5 mitoses/50 HPF)
High malignant potential	
	Group 6a (> 5cm and ≤ 10 cm, > 5 mitoses/50 HPF)
	Group 6b (> 10 cm, > 5 mitoses/50 HPF)

From [7].

*GIST* gastrointestinal stromal tumor, *HPF* high power field.

### Follow up methods and treatment

On very low- and low-risk groups according to a previous classification [8], CT was checked every 6 months during first the 2-year period, and then every year during next 3-year period. Endoscopy was performed every year for the first 5 years. For the intermediate and high-risk groups, CT was checked every 3 months for the first 2 years and then every 6 months for the next 3 years. Endoscopy was performed every year for the first 5 years.

In cases of resectable tumor recurrence, we performed additional surgery. In the other cases of unresectable tumor recurrence, imatinib treatment was started at an oral dose of 400 mg daily and was increased to 600 to 800 mg daily if the disease progressed.

### Laparoscopic surgical procedure

**Figure 1:**
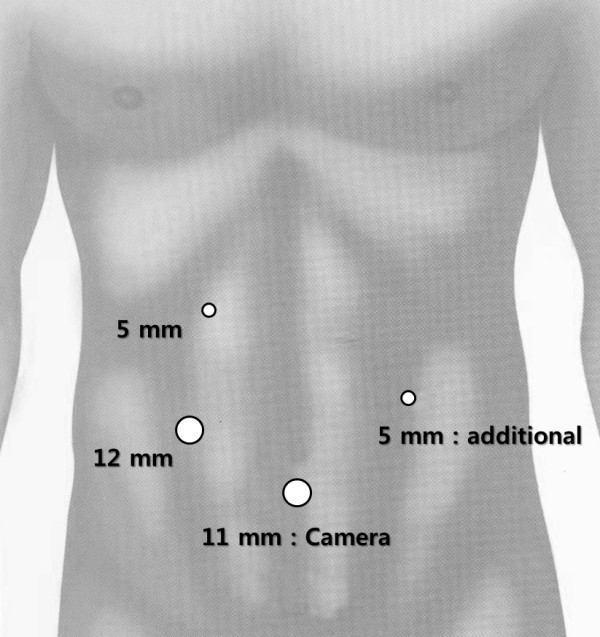
**The locations, size and site of the trocar.** Routinely, three ports (5-mm, 11-mm, and 12-mm) were used during laparoscopic surgery. Occasionally, if a further site was needed, a 5mm trocar was introduced into the left mid-abdominal area.

After the induction of general anesthesia, the patient was placed in the reverse Trendelenburg and supine position. The surgeon stood on the patient’s right, with the first assistant on the patient’s left and the camera operator on the surgeon’s right. Typically, an umbilical trocar (10 mm) was inserted using the open method. A carbon dioxide pneumoperitoneum was created using the umbilical port, and the pressure was maintained between 12 and 14 mmHg. A rigid (30 degree) laparoscope was then introduced through the umbilical port. Under laparoscopic guidance, an additional two (5-mm and 12-mm) trocars were introduced, consisting of the right subcostal and right mid-abdominal ports. Occasionally, we inserted an additional 5-mm trocar at the left mid-abdominal area (Figure 1).

After the gastric wall had been devascularized and exposed using Harmonic ACE (Ethicon Endo-Surgery, Cincinnati, OH, USA), wedge resection of the gastric wall was performed using laparoscopic stapling devices (Echelon Flex, Ethicon Endo-Surgery, Cincinnati, OH, USA).

Resected specimens were placed into an endoscopic retrieval bag and extracted via the umbilical wound. In all cases, our pathologist reported a free margin of normal gastric wall on frozen section biopsy. One closed suction drain was placed around the surgical site at the end of the procedure and trocar wounds were closed.

### Statistical analysis

Chi-square and independent *t*-tests were used to compare the clinicopathological factors of patients between the laparoscopy and open surgery group using GraphPad InStat® (version 3.06, GraphPad Software, Inc., La Jolla, CA, USA). Statistical significance was assumed for *P*-values < 0.05. Survival curves were calculated by the Kaplan-Meier method. The log-rank test was used to analyze survival differences and SPSS version 18.0 (SPSS, Chicago, Ill, USA) was used for the analysis.

## Results

### Yearly operative trends for gastric GISTs

**Figure 2:**
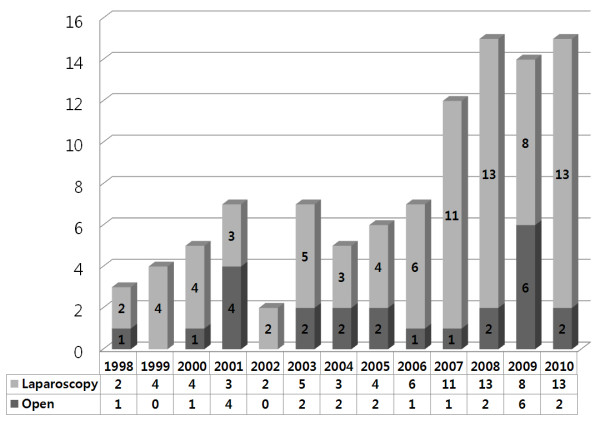
**Yearly operative trends for primary gastric gastrointestinal stromal tumor (GIST).** The numbers of laparoscopic resections has increased annually since 2007.

Figure 2 shows the operative methods (laparoscopic and open) of the patients who underwent curative resections in our institute during the period between 1998 and 2010. The numbers of laparoscopic cases has increased annually.

### Patient characteristics

Table 2 shows a summary of clinical features of the 104 patients who underwent laparoscopic and open surgery for gastric GISTs. There were 47 male and 57 female patients, with a mean age of 59.8 ± SD 10.5 years. Forty patients (38.5%) were diagnosed incidentally and were asymptomatic. Among 64 (61.5%) symptomatic patients, the most common symptom was pain. Sixty-one (58.7%), 24 (23.1%), and 19 (18.2%) of the 104 tumors were located in the upper portion, middle portion, and lower portion of the stomach, respectively. The patients were subdivided into eight groups according to Miettinen’s classification: 10 (9.6%) in group 1, 49 (47.1%) in group 2, 15 (14.4%) in group 3a, 9 (8.7%) in the group 5, 14 (13.5%) in group 6a, and 7 (6.7%) in group 6b. Laparoscopic and wedge operation were more frequently performed. Surgical margins were all observed to be free on histopathologic studies. There was no tumor rupture during surgery. Among 104 patients, there was one minor complication in a laparoscopic wedge resection. One patient who experienced delayed gastric emptying was treated with conservative care. Recurrences were noted in five patients during a median follow-up period of 49.3 (range 8.4 to 164.4) months.

**Table 2:** Characteristics and surgical outcomes of 104 patients with gastric gastrointestinal stromal tumor (GIST)

**Characteristic or outcome**	**Value**
Age, years^*^	59.8 ± 10.5
Gender	
Male/female, n (%)	47 (45.2)/57 (54.8)
Body mass index, Kg/m^2*^	24.1 ± 3.1
Symptom, n (%)	
Asymptomatic	40 (38.5)
Symptomatic	64 (61.5)
Pain	37
Dyspepsia	12
Bleeding	8
Palpable mass	2
Dizziness	5
Tumor location	
Upper/Middle/Lower, n (%)	61 (58.7)/24 (23.0)/19 (18.3)
Tumor size, cm^*^	5.1 ± 3.3
Prognostic group^†^, n (%)	
Group 1	10 (9.6)
Group 2	49 (47.1)
Group 3a	15 (14.4)
Group 3b	0 (0)
Group 4	0 (0)
Group 5	9 (8.7)
Group 6a	14 (13.5)
Group 6b	7 (6.7)
Operation, n (%)	
Laparoscopy/open	80 (76.9)/24 (23.1)
Type of resection	
Wedge resection	90 (86.5)
Partial gastrectomy	9 (8.7)
Total gastrectomy	5 (4.8)
Status of surgical margin, n (%)	
Positive/negative	0 (0)/104 (100)
Tumor rupture during operation, n (%)	
No/yes	104 (100)/0 (0)
Operation times (minutes)^*^	
Laparoscopy	91.1 ± 57.0
Open	165.8 ± 75.6
Length of hospitalization (days)^*^	
Laparoscopy	4.6 ± 2.3
Open	9.8 ± 4.1
Complication, n (%)	
Yes/no	1 (1.0)/103 (99.0)
Recurrence	
Yes/no	5 (4.8)/99 (95.2)
Median follow-up period, months, mean (range)	49.3 (8.4, 164.4)

^*^Values are mean and standard deviation; ^†^based on the Miettinen classification; *n*, number of patients.

### Clinicopathologic characteristics and postoperative outcomes of laparoscopic and open surgery for larger 5 to 10 cm tumors

To evaluate differences between laparoscopic and open surgery for larger tumor (5 to 10 cm), we compared the two groups. There were statistically significant differences in age, tumor size, tumor location, and duration of hospital stay (Table 3).

**Table 3:** Clinicopathologic characteristics and postoperative outcomes of laparoscopic and open surgery for larger 5 to 10cm tumors

	**Laparoscopy (n = 24)**	**Open (n = 14)**	***P*-value**
Age, years^*^	57.4 ± 8.1	65.9 ± 12.2	0.014
Gender, n			0.309
Male	12	4	
Female	12	10	
Body mass index, Kg/m^2*^	24.1 ± 2.9	24.0 ± 3.3	0.856
Symptom, n			0.472
No	9	3	
Yes	15	11	
Tumor size, cm^*^	6.1 ± 1.3	7.2 ± 1.7	0.035
Tumor location, n			0.041
Upper	11	8	
Middle	5	6	
Lower	8	0	
Type of resection, n			0.067
Wedge	22	9	
Partial	2	3	
Total	0	2	
Prognostic group^†^, n			
Group 2			0.239
Group 3a	5	1	
Group 4	11	4	
Group 5	2	1	
Group 6a	6	8	
Operative times, minutes^*^	119.8 ± 62.2	154.3 ± 53.5	0.092
Hospital stay, days^*^	4.8 ± 1.8	9.2 ± 3.2	< 0.001
Complication, n			1.000
No	23	14	
Yes	1	0	
Recurrence, n			0.132
No	23	11	
Yes	1	3	
Median follow-up periods, months (range)	62.6 (8.9, 164.4)	58.3 (18.8, 123.2)	0.180

^*^Values are mean and standard deviation; ^†^based on the Miettinen classification; *n* number of patients.

### Recurrence after curative resection

Table 4 shows the clinicopathologic characteristics for recurrent cases after curative resection. Tumors recurred in five patients. The recurrent cases belonged to group 5, 6a, and 6b according to Miettinen’s classification. Initial operations for these patients were open surgery in four patients and laparoscopic surgery in one patient. Tumors recurred in the peritoneum (two patients), the remnants of the stomach (one patient), the liver (one patient) and the colon (one patient). Three patients underwent reoperation, and two were treated only with imatinib mesylate. Among the patients with tumor recurrence, two have survived to date.

**Table 4:** Clinicopathologic characteristics of recurrent cases after curative resection

**N**	**Sex**	**Age**	**Size of primary tumor (cm)**	**Prognostic group**^**†**^	**Type of operation**	**Interval of operation (months)**	**Site of recurrence**	**Treatment of recurrence**	**Survival**
1	F	65	5.0	Group 5	Open	51.8	Peritoneum	Gleevec, reoperation	Dead
2	M	37	9.0	Group 6a	Open	59.7	Liver	Reoperation	Alive
3	F	64	21.0	Group 6b	Open	5.1	Colon	Gleevec	Dead
4	F	63	9.0	Group 6a	Laparoscopy	30.1	Stomach	Reoperation	Alive
5	F	81	7.6	Group 6a	Open	14.7	Peritoneum	Gleevec	Dead

*N* patient number, *F* female, *M* male; ^†^Based on the Miettinen classification.

### Survival

**Figure 3:**
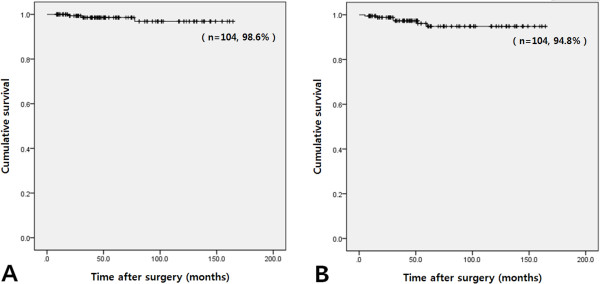
**Survival characteristics of all 104 patients.** The 5-year overall survival rate (**A**) and disease-free survival rate (**B**) were 98.6% and 94.8%, respectively.

**Figure 4:**
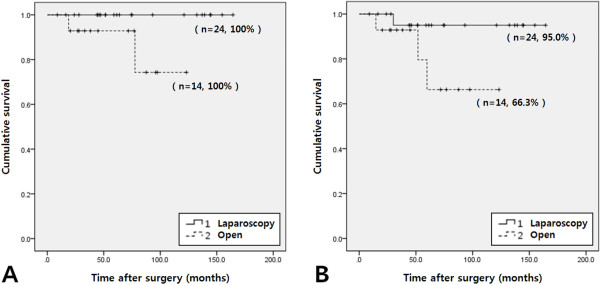
**Comparison of the 5-year overall and disease-free survival rate between laparoscopic and open surgery for larger 5 to 10cm tumors.** The 5-year overall survival rates (**A**) and disease-free survival rates (**B**) were 100% and 92.9% (*P* = 0.067) and 95.0% and 66.3% (*P* = 0.083), in the laparoscopic and open surgery groups respectively

The 5-year overall and disease-free survival rates of gastric GISTs were 98.6% and 94.8%, respectively (Figure 3). Comparison of cases of laparoscopic and open surgery for large tumors (5 to 10 cm) showed that there were no statistically significant differences in 5-year overall and disease-free survival rates between the two groups (*P* = 0.067 and 0.083, respectively) (Figure 4).

## Discussion

Surgical resection with negative margins without lymphadenectomy has been the treatment of choice of gastric GISTs up to now [1]. Histologically, a 1 to 2 cm margin has been thought to be necessary for adequate resection [9,10]. However, more recently, DeMatteo *et al*. [11] said that tumor size and negative microscopic surgical margins did not determine the survival. It is therefore accepted that the surgical goal should be complete resection with gross negative margins only [3,11]. Given this, wedge resection has been advocated by many investigators for the majority of gastric GISTs [3,10,11]. Currently, gastric GISTs are viewed as a good indication for laparoscopic resection. Moreover, the development of laparoscopic stapling devices and surgical techniques has made laparoscopic wedge resection an attractive alternative to conventional open surgery [12]. In this study, laparoscopic surgery has been performed with an annually increasing tendency, and wedge resection was more commonly advocated. This shows that laparoscopic wedge resection has become the mainstay of treatment for gastric GIST.

Recent reports from the National Comprehensive Cancer Network (NCCN) GIST Task Force and the GIST Consensus Conference under the auspices of The European Society for Medical Oncology (ESMO) state that laparoscopic or laparoscopic-assisted resection may be used for small gastric GISTs (that is, those < 2 cm in size) [6]. However, Ronellenfitsch *et al*. [13] stated that the tumor size did not determine the feasibility of laparoscopic wedge resection, and the location of the gastric GISTs did not directly affect the indication for laparoscopic wedge resection. Whereas Yang *et al*. [14] reported on the performance of laparoscopic wedge resection for tumors less than 6 cm in diameter, Ronellenfitsch *et al*. [13] and Huguet *et al*. [15] reported its feasibility for tumors bigger than 10 cm in diameter. The Japanese clinical practice guidelines for GIST suggest that laparoscopic resection of gastric GISTs smaller than 5 cm appears safe when performed by a skillful surgeon who is thoroughly familiar with the neoplastic characteristics of gastric GISTs [16]. Before 2005, in our institute we performed open surgery for tumors bigger than 5 cm and for those located at the cardia. As our experience has increased, we have been performing laparoscopic surgery on tumors smaller than 10 cm regardless of their location.

We classified the 38 patients who had large tumors (5 to 10 cm) into those who received laparoscopic versus open surgery. Although there were statistically significant differences in age, tumor size, and tumor location, we thought that these variables were not considered to be factors that were comparable between laparoscopic and open surgery. From the point of view of the merit of laparoscopic surgery, the length of hospitalization was statistically shorter in laparoscopic surgery than in open surgery. Moreover, the operation time was shorter in laparoscopic surgery, although there was no statistical difference. In terms of survival rates, there were no statistical differences in overall and disease-free survival rates, although the survival graphs appeared to be different.

In the case of gastric GISTs bigger than 10 cm, surgeons were concerned about the operative methods of laparoscopic versus open surgery. The merits of laparoscopic surgery included lesser degree of pain, smaller wound size, shorter hospital stay, and earlier recovery. However, in order to safely retrieve a mass bigger than 10 cm, a larger wound incision was needed, as in open surgery. Moreover, laparoscopic surgical techniques became more difficult in cases with bigger gastric GISTs, and there was a possibility that tumor cells would spread due to the rupture of the capsules. Therefore, with bigger tumors, special attention should be paid to the prevention of capsular rupture. It should also be emphasized that careful laparoscopic evaluation of the tumor size, and its characteristics in terms of the possibility of capsular rupture during further manipulations, should be performed, giving timely conversion to the open method whenever necessary. In our series, for the prevention of tumor spread during laparoscopic surgery, we tried to grasp the stomach and normal tissues around the tumor. In our study, there were seven patients with tumors bigger than 10 cm, three of whom underwent laparoscopic surgery, while four underwent open surgery. In addition, there were no capsular ruptures in the three patients who had laparoscopic surgery.

The recurrence rate after surgery in reported series ranges from 17 to 24% [17,18]. In recurrent gastric GISTs, some reports demonstrated that a combination of surgery and targeted therapy may reduce the development of recurrence or decrease the risk of disease progression [19,20]. Although most of our patients who underwent surgical resection were at very low, or low malignant potential (74/104, 71.2%), we had a lower recurrence rate in our series compared to other reports [17,18]*.* We experienced five cases (5/104, 4.8%) of recurrence, with a median follow-up time of 49.3 months (range, 8.4 to 164.4 months) after surgical resection for gastric GISTs. Three patients underwent reoperation, and two were treated with imatinib mesylate. Unfortunately, none of the patients responded to imatinib mesylate, and two patients who underwent surgical management are currently living.

Although this was a retrospective research study of laparoscopic and open surgery for gastric GIST, and large tumors 5 to 10 cm in size, and although it was not a case-matched study of laparoscopic and open surgery, it provides a basic guideline to determine the size-related indication for laparoscopic surgery for gastric GIST. A prospective randomized controlled study of tumors larger than 5 cm is necessary.

## Conclusion

The clinical outcomes of gastric GISTs with very low or low malignant potential were excellent. The group of patients who had gastric GIST with high malignant potential showed an increased recurrence rate and less favorable survival rates, which merits careful attention. In terms of the operative method, laparoscopic surgery was an effective oncological treatment for gastric GIST. Although this was a retrospective, research study in a single institute, it is thought that laparoscopic surgery would be a good alternative to open surgery for the treatment of large gastric GIST bigger than 5 cm in size.

## Abbreviations

EGDS: Esophagogastroduodenoscopy; EUS: Endoscopic ultrasonography; GIST: Gastrointestinal stromal tumor; HPF: High-power field; NCCN: National Comprehensive Cancer Network; NIH: National Institutes of Health; ESMO: European Society for Medical Oncology.

## Competing interests

The authors have no competing interests to declare.

## Authors’ contributions

Kim KH, Kim MC carried out data collection. Kim SJ did pathological re-examination and diagnosis. Jung GJ, Jang JS, and Kwon HC helped draft the manuscript. All authors read and approved the final manuscript.

## References

[B1] Nowain A Bhakta H Pais S Kanel G Verma S Gastrointestinal stromal tumors: clinical profile, pathogenesis, treatment strategies and prognosis J Gastroentrol Hepatol 2005 20 818 824 10.1111/j.1440-1746.2005.03720.x 15946127

[B2] Mochizuki Y Kodera Y Fujiwara M Ito S Yamamura Y Sawaki A Yamao K Kato T Laparoscopic wedge resection for gastrointestinal stromal tumors of the stomach: initial experience Surg Today 2006 36 341 347 10.1007/s00595-005-3164-7 16554991

[B3] Heinrich MC Corless CL Gastric GI stromal tumors (GISTs): the role of surgery in the era of targeted therapy J Surg Oncol 2005 90 195 207 10.1002/jso.20230 15895440

[B4] Demetri GD Benjamin RS Blanke CD Blay JY Casali P Choi H Corless CL Debiec-Rychter M DeMatteo RP Ettinger DS Fisher GA Fletcher CD Gronchi A Hohenberger P Hughes M Joensuu H Judson I Le Cesne A Maki RG Morse M Pappo AS Pisters PW Raut CP Reichardt P Tyler DS Van den Abbeele AD von Mehren M Wayne JD Zalcberg J NCCN Task Force NCCN Task Force report: management of patients with gastrointestinal stromal tumor (GIST)--update of the NCCN clinical practice guidelines J Natl Compr Canc Netw 2007 Suppl 2 S1 S29 17624289

[B5] Otani Y Furukawa T Yoshida M Saikawa Y Wada N Ueda M Kubota T Mukai M Kameyama K Sugino Y Kumai K Kitajima M Operative indications for relatively small (2-5cm) gastrointestinal stromal tumor of the stomach based on analysis of 60 operated cases Surgery 2006 139 484 492 10.1016/j.surg.2005.08.011 16627057

[B6] Blay JY Bonvalot S Casali P Choi H Debiec-Richter M Dei Tos AP Emile JF Gronchi A Hogendoorn PC Joensuu H Le Cesne A Mac Clure J Maurel J Nupponen N Ray-Coquard I Reichardt P Sciot R Stroobants S van Glabbeke M van Oosterom A Demetri GD GIST consensus meeting panelists Consensus meeting for the management of gastrointestinal stromal tumors. Report of the GIST Consensus Conference of 20–21 March 2004, under the auspices of ESMO Ann Oncol 2005 16 566 578 10.1093/annonc/mdi127 15781488

[B7] Miettinen M Sobin LH Lasota J Gastrointestinal stromal tumors of the stomach: a clinicopathologic, immunohistochemical, and molecular genetic study of 1765 cases with long-term follow-up Am J Surg Pathol 2005 29 52 68 10.1097/01.pas.0000146010.92933.de 15613856

[B8] Fletcher CD Berman JJ Corless C Gorstein F Lasota J Longley BJ Miettinen M O’Leary TJ Remotti H Rubin BP Shmookler B Sobin LH Weiss SW Diagnosis of gastrointestinal stromal tumors: a consensus approach Hum Pathol 2002 33 459 465 10.1053/hupa.2002.123545 12094370

[B9] Matthews BD Walsh RM Kercher KW Sing RF Pratt BL Answini GA Heniford BT Laproscopic vs open resection of gastric stromal tumors Surg Endosc 2002 16 803 807 10.1007/s00464-001-8319-z 11997826

[B10] Rosen MJ Heniford BT Endoluminal gastric surgery: the modern era of minimally invasive surgery Surg Clin North Am 2005 85 989 1007 10.1016/j.suc.2005.05.010 16139032

[B11] DeMatteo RP Lewis JJ Leung D Two hundred gastrointestinal stromal tumors: recurrence patterns and prognostic factors for survival Ann Surg 2000 231 51 58 10.1097/00000658-200001000-00008 10636102 PMC1420965

[B12] Hyung WJ Lim JS Cheong JH Kim J Choi SH Noh SH Laparoscopic resection of a huge intraluminal gastric submucosal tumor located in the anterior wall: eversion method J Surg Oncol 2005 89 95 98 10.1002/jso.20195 15660368

[B13] Ronellenfitsch U Staiger W Kahler G Strobel P Schwarzbach M Hohenberger P Perioperative and oncological outcome of laparoscopic resection of gastrointestinal stromal tumour (GIST) of the stomach Diagn Ther Endosc 2009 2009 286138 19343179 10.1155/2009/286138 PMC2662319

[B14] Yang HK Park DJ Lee HJ Kim HH Kim WH Lee KU Clinicopathologic characteristics of gastrointestinal stromal tumor of the stomach Hepatogastroenterology 2008 55 1925 1930 19102424

[B15] Huguet KL Rush RM Jr Tessier DJ Schlinkert RT Hinder RA Grinberg GG Kendrick ML Harold KL Laparoscopic gastric gastrointestinal stromal tumor resection: the mayo clinic experience Arch Surg 2008 143 587 590 10.1001/archsurg.143.6.587 18559753

[B16] Nishida T Hirota S Yanagisawa A Sugino Y Minami M Yamamura Y Otani Y Shimada Y Takahashi F Kubota T GIST Guideline Subcommittee Clinical practice guidelines for gastrointestinal stromal tumor (GIST) in Japan: English version Int J Clin Oncol 2008 13 416 430 10.1007/s10147-008-0798-7 18946752

[B17] Demetri GD von Mehren M Blanke CD Van den Abbeele AD Eisenberg B Roberts PJ Heinrich MC Tuveson DA Singer S Janicek M Fletcher JA Silverman SG Silberman SL Capdeville R Kiese B Peng B Dimitrijevic S Druker BJ Corless C Fletcher CD Joensuu H Efficacy and safety of imatinib mesylate in advanced gastrointestinal stromal tumors N Engl J Med 2002 347 472 480 10.1056/NEJMoa020461 12181401

[B18] Iesalnieks I Rümmele P Dietmaier W Jantsch T Zülke C Schlitt HJ Hofstädter F Anthuber M Factors associated with disease progression in patients with gastrointestinal stromal tumors in the pre-imatinib era Am J Clin Pathol 2005 124 740 748 10.1309/AKK3VFF610CWM566 16203282

[B19] van der Zwan SM DeMatteo RP Gastrointestinal stromal tumor: 5 years later Cancer 2005 104 1781 1788 10.1002/cncr.21419 16136600

[B20] An JY Choi MG Noh JH Sohn TS Kang WK Park CK Kim S Gastric GIST: a single institutional retrospective experience with surgical treatment for primary disease Eur J Surg Oncol 2007 33 1030 1035 10.1016/j.ejso.2007.02.009 17428635

